# Women's workplace transitions in saudi arabia following the driving reform: An ordered logit analysis

**DOI:** 10.1371/journal.pone.0354251

**Published:** 2026-08-31

**Authors:** Ghada Alturif, Wafaa Saleh

**Affiliations:** 1 Department of Social Work, College of Humanities and Social Sciences, Princess Nourah bint Abdulrahman University, Riyadh, Saudi Arabia; 2 Transport Research Institute, School of Computing, Engineering and the Built Environment, Edinburgh Napier University, Edinburgh, United Kingdom; Western Sydney University, AUSTRALIA

## Abstract

**Background:**

The removal of the driving ban for women in Saudi Arabia in 2018 represented a landmark socio-economic reform under Vision 2030, with important implications for women’s mobility and career trajectories. However, limited empirical evidence exists on the relationship between this reform and women’s employment-related mobility, particularly workplace transitions, using applied modelling approaches and multi-level analytical frameworks.

**Aim:**

This study applies an ordered logit modelling approach to examine the association between women’s driving rights and employment-related mobility, specifically assessing the likelihood of workplace transitions among Saudi women. The analysis is structured using the Social Ecological Model (SEM) to capture the multi-level factors shaping women's mobility and workplace transitions.

**Methodology:**

This quantitative survey study utilised primary data collected from 890 Saudi women across diverse regions, socioeconomic groups, and employment categories. Official secondary data from national statistical and policy sources were used to contextualise and interpret the findings at the institutional and policy levels rather than being directly incorporated into the econometric model. Descriptive statistics, including means, standard deviations, and cross-tabulations, were used to characterise patterns of workplace mobility. An ordered logit model was then employed to estimate the likelihood of workplace transitions. The final model identified licence ownership, age, income, and marital status as significant predictors.

**Results:**

Possessing a driver's licence was positively associated with workplace transitions and more than doubled the odds of employment-related mobility (β = 0.80, OR = 2.23, 95% CI: 1.66–2.99, p < 0.001). Younger age and higher income were associated with a greater likelihood of workplace transitions, whereas married women reported lower levels of employment-related mobility. At the policy level, women widely perceived Vision 2030 reforms as facilitating greater autonomy, empowerment, and workplace mobility.

**Conclusions:**

The findings suggest that driving access is positively associated with greater workplace mobility and labour market flexibility among Saudi women. However, these associations vary across age groups, marital status, and income levels. By focusing on workplace transitions rather than labour market entry, this study provides an applied modelling framework for examining how mobility reforms relate to employment-related mobility and career opportunities, offering transferable insights for other socio-economic contexts undergoing gender and mobility transformations.

## 1. Introduction

Historically, the Saudi labour market has been shaped by deep-rooted social norms, legal restrictions, and gender-specific mobility constraints that limited women’s participation in the workforce. Among the most symbolic and practical barriers was the prohibition on women driving, which remained in place until June 2018. The inability to independently navigate public and private spaces constrained women’s access to employment, especially in regions with limited public transportation infrastructure or where reliance on male guardians or hired drivers was both financially and socially restrictive. The lifting of the driving ban coincided with a broader package of reforms introduced under Vision 2030 aimed at increasing women's economic participation and advancing social inclusion. These reforms included labour market initiatives promoting the employment of Saudi nationals (Saudization), changes to guardianship regulations, expansion of employment opportunities in previously restricted sectors, improvements in female educational attainment, and evolving social attitudes towards women's public roles. Consequently, improvements in women's labour market outcomes cannot be attributed solely to driving access. Rather, the removal of the driving ban should be understood as one important structural reform operating alongside these wider institutional and societal changes.

From an economic perspective, independent driving may influence women's labour market behaviour through several mechanisms. First, it reduces the direct and indirect costs of commuting by eliminating or reducing expenditure on private drivers and lowering dependence on family members for transport. Second, it increases temporal flexibility, enabling women to better coordinate work schedules with family responsibilities and to respond to employment opportunities requiring irregular or longer working hours. Third, it expands the spatial range of accessible jobs, particularly in areas where public transport services remain limited. Together, these mechanisms may facilitate workplace transitions, improve access to more desirable employment opportunities, and increase career mobility.

Recent statistics reinforce the scale of change. According to the General Authority for Statistics (2023), female labour force participation rose from 17.7% in 2016 to 37% by 2023, coinciding with the lifting of the driving ban and broader Vision 2030 initiatives. Female unemployment fell from 33% in 2016 to 15.4% in 2023, while more than 180,000 Saudi women obtained driving licences by late 2021. Car ownership among women has also increased steadily, reflecting a shift toward greater independence. However, the benefits have been uneven: urban centres such as Riyadh and Jeddah show 65–70% higher rates of female employment and car ownership than rural regions, highlighting regional disparities in access to mobility-enabled opportunities.

It is important to distinguish between different labour market outcomes that may be associated with enhanced mobility. Labour force participation refers to entering or remaining in the workforce; workplace transitions describe movement between jobs, workplaces, or employment settings; occupational mobility concerns shifts in occupational status or career progression; job quality encompasses dimensions such as wages, working conditions, and satisfaction; while perceptions of empowerment capture subjective experiences of autonomy and agency. Although these outcomes are related, they are conceptually distinct. The present study focuses specifically on employment-related mobility through workplace transitions rather than labour market participation more broadly.

To conceptualize these dynamics, this study employs the Social Ecological Model (SEM), which frames behaviour and outcomes as products of interacting levels of influence. At the individual level, women’s demographic and socioeconomic characteristics (e.g., age, education, income, marital status) shape their capacity to benefit from driving. At the interpersonal level, family roles and household dependencies influence whether and how women can act on mobility rights. At the institutional/community level, workplace structures, social norms, and local infrastructure determine the accessibility and quality of opportunities. Finally, at the policy level, the removal of the driving ban and Vision 2030 reforms act as structural enablers of inclusion and empowerment. Integrating SEM with econometric modelling allows this study to capture both the micro-level determinants of women’s job mobility and workplace transitions and the broader structural context of reform.

The aim of this paper is to examine the association between women's driving access and employment-related mobility, operationalised as workplace transitions among Saudi women, while considering how demographic and socioeconomic factors shape these relationships.

By combining descriptive analysis with ordered logit modelling, the study provides applied evidence of how mobility reform has reshaped labour market trajectories, while offering policy-relevant insights into the pursuit of gender equity and economic diversification under Vision 2030. The study is guided by two research questions:

Q1: To what extent does gaining the ability to drive influence the likelihood of Saudi women changing their workplace or job role?
Q2: How do socioeconomic and interpersonal factors (e.g., income, marital status, and number of dependents) interact with driving ability to shape workplace transitions and employment-related mobility among Saudi women?

## 2. Literature review

The role of female mobility in shaping labour market outcomes has been widely documented across different national contexts, particularly where social norms, transport disadvantage, and institutional barriers constrain women’s economic participation. Access to transport influences not only whether women can reach employment, but also job search behaviour, commuting distance, occupational choice, job quality, and career progression [1–3]. Mobility also carries symbolic value, especially in gender-segregated or patriarchal contexts, where independent movement can represent autonomy, agency, and social empowerment [4–6].

International evidence shows that women’s labour market outcomes are strongly shaped by mobility constraints and commuting conditions. Earlier comparative work demonstrated that women’s labour force participation is shaped by broader social contexts and gender-role expectations [5,7]. More recent evidence from South Asia further confirms that mobility barriers restrict women’s job search and labour supply. Field and Vyborny [8,9] show that improving women’s mobility can increase labour supply and expand access to work opportunities, while Shoaib et al. [10] demonstrate that transport-related constraints continue to limit women’s access to employment in Lahore, Pakistan. These findings suggest that the relationship between mobility and labour outcomes is not purely individual, but is mediated by household responsibilities, social norms, transport systems, and institutional conditions.

In Saudi Arabia, women’s mobility and employment have historically been shaped by legal restrictions, social expectations, and limited independent transport options. Prior to the lifting of the driving ban in 2018, many women relied on male relatives or paid drivers, creating financial, temporal, and social constraints on access to employment [11,12]. Studies on women’s work in Saudi Arabia identify persistent barriers to career advancement, including organisational constraints, gender norms, limited mobility, and family responsibilities [13–18]. These barriers were especially significant in areas with limited public transport provision, where independent driving could reduce commuting costs and expand access to workplaces [11].

The lifting of the driving ban occurred alongside wider Vision 2030 reforms aimed at increasing women’s economic participation and national diversification [19,20]. Saudi-focused studies suggest that the reform contributed to changing perceptions of women’s mobility, employment, and empowerment [21–26]. Macias-Alonso et al. [22], for example, highlight the gendered mobility implications of the reform, while Saleh and Malibari [23] examine women’s perceptions of equality and empowerment in relation to Vision 2030. See also further discussions on the necessity of sustained reforms and gender-sensitive policies, outlined practical tools for achieving social empowerment, which supports the role of mobility in enhancing women’s agency, and the financial burden of relying on private drivers, emphasizing the economic rationale for women’s self-mobility [27–30]. Recent evidence by Abou Daher et al. [31] provides important empirical support for employment responses following the lifting of the driving ban. However, much of the existing literature focuses on labour force participation, empowerment, leadership, or broad employment outcomes rather than workplace transitions and job mobility among women already engaged in employment.

The Saudi case should also be understood within the wider regional labour market context. In the MENA region, women’s employment is shaped by both firm-level and national-level factors, including institutional structures, labour market segmentation, and gendered expectations [32]. Legal and policy reforms have created new opportunities for women in Saudi Arabia, but their effects are conditioned by workplace practices, family expectations, and social norms [25,33–35]. This is consistent with international research showing that mobility reforms alone may be insufficient unless accompanied by institutional and social support mechanisms [36].

The Social Ecological Model (SEM) provides a useful framework for examining these multi-level influences. SEM emphasises that individual behaviour and outcomes are shaped by interacting systems at the individual, interpersonal, institutional, community, and policy levels [37]. In the context of women’s workplace transitions, individual factors such as age, education, income, and licence ownership affect the capacity to benefit from driving. Interpersonal factors, including marital status, dependents, and household responsibilities, may enable or constrain women’s ability to act on mobility rights. Institutional and community factors, such as workplace flexibility, employment sector, and regional transport infrastructure, shape whether mobility translates into actual workplace change. Policy-level reforms, including the lifting of the driving ban and Vision 2030 initiatives, create the broader structural conditions within which these outcomes unfold [10,33].

Methodologically, existing studies on women’s mobility and empowerment have used qualitative, descriptive, and policy-oriented approaches, but fewer have applied econometric models to examine workplace transitions as an ordinal outcome. Ordered logit models are appropriate for analysing ranked dependent variables where outcomes reflect increasing levels of change or intensity [38]. Brant [39] also provides methodological guidance for assessing proportionality in proportional odds models. In the present study, the ordered logit model is used to examine the likelihood of different levels of workplace transition associated with driving access and socio-demographic characteristics.

Despite growing scholarship on women’s mobility and employment in Saudi Arabia, three important gaps remain. First, existing studies largely focus on labour force participation, empowerment perceptions, leadership, or general employment outcomes, rather than employment-related mobility in the form of workplace transitions. Second, many studies examine individual or household factors without fully integrating interpersonal, institutional, community, and policy-level influences. Third, there remains limited applied modelling evidence on how women’s driving access is associated with job mobility and workplace transitions in the Saudi context.

Accordingly, this study contributes to the literature in three ways. First, it shifts the focus from employment participation to employment-related mobility by examining workplace transitions following the lifting of the driving ban. Second, it applies the Social Ecological Model to provide a multi-level understanding of how individual, interpersonal, institutional, and policy factors shape women’s workplace mobility. Third, by integrating SEM with ordered logit modelling using survey data from 890 Saudi women, the study provides an applied empirical framework for examining how mobility reforms under Vision 2030 are associated with measurable workplace transition outcomes.

## 3. Methodology

### 3.1. Study area and analytical framework

The study is situated in the Kingdom of Saudi Arabia (KSA), a country undergoing rapid social and economic transformation, particularly in relation to women’s roles in the workforce. Historically, the Saudi labour market was shaped by gender-specific restrictions and mobility constraints that limited women’s participation and access to diverse employment opportunities. The lifting of the driving ban in June 2018 constituted a landmark reform, directly enhancing female mobility and opening new avenues for employment, social empowerment, and household decision-making.

To examine these changes within an applied, multi-level framework, this study adopts the Social Ecological Model (SEM) as its guiding analytical lens. SEM conceptualises women’s mobility and labour market participation as outcomes of interrelated systems operating at four levels:

- Individual level: socio-demographic characteristics such as age, education, income, and marital status that influence the ability to utilise mobility for job mobility and workplace transitions.
- Interpersonal level: household and family dynamics, including dependence on male guardians or paid drivers, as well as family responsibilities that may facilitate or restrict women’s job mobility.
- Institutional/community level: workplace practices, employment sectoral differences, and regional variations in infrastructure and public transport that determine how mobility opportunities are realised.
- Policy level: national reforms, including the removal of the driving ban and broader Vision 2030 initiatives, which act as structural enablers of gender inclusion in the labour market.

Embedding SEM in the applied design ensures that the analysis goes beyond measuring the direct impact of driving on job mobility. It should be noted however that the final ordered logit model in this research retained only variables that remained statistically significant and theoretically relevant after model specification.

### 3.2. Data collection

Primary data were collected through a structured questionnaire administered to a sample of 890 Saudi women spanning a wide range of employment categories, from unemployed and low-skilled positions to mid- and high-level professional roles. The questionnaire was carefully designed based on the literature review and prior empirical studies, with explicit integration of the Social Ecological Model (SEM) dimensions:

- Individual level: demographic and socioeconomic indicators (age, education, marital status, family size, monthly income, driving license ownership, years of driving experience).
- Interpersonal level: reliance on family members or hired drivers, household responsibilities, and family attitudes toward women’s driving.
- Institutional/community level: employment sector, workplace flexibility, job opportunities, and regional infrastructure.
- Policy level: perceptions of Vision 2030 reforms, government support for women’s empowerment, and the influence of structural mobility policies.

Inclusion criteria required participants to be Saudi women aged 18 years or older who were residing in Saudi Arabia during the study period and willing to provide verbal informed consent. Because the study focused on employment-related mobility following the lifting of the driving ban, women from all employment categories, including unemployed participants, were eligible to participate.

Data collection was conducted over a four-month period, from Monday 13 March to 14 July 2023, across multiple geographic locations to ensure broad representation of the Kingdom. The online questionnaire targeted Saudi women from diverse regions, professions, and socioeconomic backgrounds. A non-probability sampling strategy was adopted, combining purposive and convenience sampling techniques. The survey link was disseminated through professional networks, social media platforms, and community contacts to maximise participation from Saudi women across different regions, age groups, employment categories, and socioeconomic backgrounds. Although efforts were made to achieve broad representation, the sampling approach does not permit claims of national representativeness and may have favoured women who were digitally connected and more willing to participate in online surveys. Also, the use of an online survey may have introduced selection bias, as women with limited internet access, lower levels of digital literacy, older age groups, or those living in more remote areas may have been less likely to participate. The survey was disseminated through digital platforms, including email, WhatsApp, and Twitter, to maximise reach and inclusivity, with particular emphasis on engaging women in both urban and suburban settings. Verbal informed consent was obtained from all participants. Prior to participation, all participants received a detailed explanation of the study objectives, procedures, potential risks and benefits, confidentiality measures, and their right to withdraw at any time without consequence. Verbal informed consent was obtained from each participant before the interview commenced. The interviewer documented that consent had been obtained by recording this in the study data collection record before proceeding with the interview. Where applicable, the verbal consent procedure was witnessed by a member of the research team. The use of verbal informed consent, including the method of documentation, was reviewed and approved by the Institutional Review Board (IRB)/Research Ethics Committee as part of the approved study protocol.

Consequently, certain segments of the population may be underrepresented, and therefore the findings should be interpreted as indicative associations rather than nationally representative estimates of Saudi women. These limitations should be considered when interpreting and generalising the findings.

The survey was developed using Google Forms and included a combination of closed-ended and three-point Likert-scale questions (Disagree, Don't Know, Agree). The three-point format was selected to simplify response options, reduce respondent burden, and facilitate completion among participants with diverse educational backgrounds. While a scale with a larger number of response categories may capture greater nuance in perceptions, the simpler format was considered appropriate for this study given its broad target population and exploratory focus. Prior to full deployment, the instrument underwent pilot testing to ensure clarity, validity, and reliability. The online format was chosen for its efficiency, cost-effectiveness, and suitability for safely engaging a large and geographically dispersed population, particularly in light of cultural norms and logistical constraints affecting in-person data collection. A total of 890 valid responses were obtained and included in the final analysis.

The questionnaire included the following sections:

Section A: Demographic characteristics (e.g., age, education, marital status)
Section B: Driving and mobility characteristics (e.g., licence ownership, car ownership)
Section C: Employment and workplace transitions
Section D: Family and interpersonal influences
Section E: Perceptions of Vision 2030 and mobility reforms
Section F: Attitudinal statements (27 items measured on a three-point Likert scale)

The 27 attitudinal statements designed to capture perceptions of how the lifting of the driving ban affected job mobility and labour market dynamics. In addition to primary data, secondary data from the General Authority for Statistics (GASTAT) [33], Vision 2030 reports, and national labour market assessments were incorporated to strengthen contextual interpretation and policy-level analysis. A total of 890 valid responses were collected and retained for analysis.

## 4. Analysis

The analytical strategy followed a two-stage process consistent with applied modelling in social sciences: descriptive exploration followed by inferential econometric modelling. Questionnaires with substantial missing information were excluded during data cleaning. Responses with minor item-level missing data were minimal and were handled using listwise deletion in the regression analysis. Consequently, the final analytical sample consisted of 890 complete cases. Descriptive statistics, including means, standard deviations, and frequencies, were computed to summarise general trends. Cross-tabulations were used to explore relationships between categorical variables such as marital status and driving ability, or education and employment sector. To align with the SEM framework, descriptive results were further stratified by level of influence (individual, interpersonal, institutional/community, and policy) to reveal patterns of heterogeneity across different socio-ecological contexts.

Ordered Logit Model (OLM)

To quantify the impact of mobility on workplace transitions, an Ordered Logit Model (OLM) was specified. The dependent variable measured the degree of workplace change due to driving, coded as an ordinal outcome:

- 1 = No change
- 2 = Some change (e.g., timing, job type, commuting distance)
- 3 = Significant change (e.g., sector change, promotion, or entirely new job).

The ordered logit model (OLM) expression is given by:


$$\begin{array}{c} \mathrm{Logit}\left[P(Y\leq j)\right]=\alpha_{j}-\beta_{1}X_{1}-\beta_{2}X_{2}-\cdots -\beta_{k}X_{k} \end{array}$$ (1)

Y is each ordered category level or the outcome variable (job change level)

j = cutoff thresholds

𝑋_𝑘_ = independent variables

𝛼_𝑗_ = threshold parameters

𝛽_𝑘_ = estimated coefficients

Consistent with the study objectives and the availability of complete analytical outputs, the final ordered logit specification included age, monthly income, marital status, and driver's licence ownership. These variables were selected because they represent key individual and interpersonal dimensions of the Social Ecological Model and have been identified in previous literature as important determinants of women's employment-related mobility. Other variables considered within the broader SEM framework were examined descriptively and used to contextualise the findings but were not included in the final regression model presented in this study. Secondary controls were integrated from GASTAT and Vision 2030 reports to contextualise institutional and policy-level dynamics. In this case, results are not only statistically robust but also socio-ecologically interpretable. For instance, individual-level variables (e.g., age and income) reveal how personal characteristics enable or constrain workplace mobility. This multi-level analytical design ensures that the study contributes both methodologically and practically to the state of the art on women mobility,job mobility and employment transition and workplace transitions in the Saudi Arabia.

## 5. Results

The study sample consisted of 890 Saudi female respondents representing a diverse range of employment categories, from unemployed and low-skilled jobs to high-level professional positions. Table 1 summarizes the socio-demographic characteristics of the respondents.

**Table 1:** Socio-Demographic Characteristics of Respondents (N = 890).

Variable	Categories	Frequency (n)	Percentage (%)	Mean	Std. Deviation (SD)
Age Group (years)	18-25	210	23.6	34.7	8.9
	26-35	350	39.3		
	36-45	200	22.5		
	46+	130	14.6		
Marital Status	Single	300	33.7		
	Married	520	58.4		
	Divorced/Widowed	70	7.9		
Monthly Family Income (SAR)	<5,000	150	16.9	12,500	8,200
	5,000 - 10,000	320	36.0		
	>10,000	420	47.1		
Employment Category	Unemployed	180	20.2		
	Low-skilled jobs	250	28.1		
	Mid-skilled jobs	280	31.5		
	High-skilled/professional jobs	180	20.2		

The socio-demographic distribution highlights key individual-level factors within the SEM framework. Respondents were primarily in the younger and middle age groups (62.9% aged 18–35), with a majority being married (58.4%). Almost half of the respondents reported household monthly incomes above SAR 10,000, while employment categories were broadly distributed across unemployed, low-skilled, mid-skilled, and professional groups. These baseline figures illustrate the diversity of women’s positions in the labour market and provide the foundation for assessing how mobility influences transitions. Respondents’ attitudes toward the impact of enabling women to drive on their employment and empowerment were assessed using a three-point Likert scale. Table 2 presents a subset of five attitudinal statements that were selected because they most directly reflect the study objectives and the four levels of the Social Ecological Model. The remaining items are available from the authors upon request and were excluded from the main manuscript to improve readability.

**Table 2:** Descriptive Statistics for Key Attitudinal Statements.

Attitudinal Statement	Mean	Std. Deviation	% Agree	% Don’t Know	% Disagree
Driving facilitates women’s mobility to work	2.87	0.42	90.4	6.3	3.2
Driving shortens commuting time	2.81	0.49	85.2	10.6	4.2
Driving encourages women’s participation in the labour market	2.79	0.51	83.1	12.4	4.4
Driving expands job opportunities for women	2.80	0.49	83.6	12.7	3.8
Vision 2030 has empowered women economically and socially	2.86	0.39	88.7	9.4	1.9

Attitudinal responses strongly affirmed the enabling role of driving across multiple SEM levels. At the individual level, 90.4% agreed that driving facilitates mobility to work, while 85.2% reported reduced commuting time, directly linking mobility to personal autonomy and efficiency. At the interpersonal level, women noted that driving reduced dependency on male guardians or hired drivers, supporting greater decision-making independence within households. At the institutional/community level, over 83% agreed that driving expands job opportunities and encourages labour market participation, indicating improved access to workplaces across sectors and regions. Finally, at the policy level, nearly 89% recognized Vision 2030 as a catalyst for women’s empowerment, demonstrating alignment between national reforms and individual perceptions of opportunity. The relationship between employment category and the reported likelihood of changing workplace due to driving mobility was also examined (Table 3).

**Table 3:** Employment Category by Likelihood of Changing Workplace.

Employment Category	Likely to Change Workplace (%)	Not Likely to Change (%)	Total (n)
Unemployed	15.6	84.4	180
Low-skilled Jobs	38.0	62.0	250
Mid-skilled Jobs	47.5	52.5	280
High-skilled/Professional	56.1	43.9	180

Table 3 presents descriptive differences in the reported likelihood of workplace transitions across employment categories. Women employed in high-skilled and professional occupations more frequently reported a likelihood of workplace change (56.1%) than unemployed respondents (15.6%). These patterns may reflect differences in career structures, advancement opportunities, and occupational mobility across employment sectors. However, because these findings are descriptive, they should not be interpreted as evidence that employment category directly causes workplace transitions.

An Ordered Logit Model (OLM) was applied, where the dependent variable captured the likelihood of workplace change due to driving (Table 4).

**Table 4:** Categories for the Dependent Variable (Ordered Logit Model).

Category Code	Description	Interpretation
1	Unlikely	Respondent is unlikely to change workplace
2	Neutral / Unsure	Respondent is uncertain or neutral about changing workplace
3	Likely	Respondent is likely to change workplace

Table 5 presents the results of the ordered logit model estimating the likelihood of workplace change due to women’s ability to drive. Several variables were considered for model specification based on the SEM framework. However, the final model presented includes variables retained in the selected specification due to statistical significance, model fit considerations, and data completeness. The final ordered logit model retained only variables that remained statistically significant and theoretically relevant after model specification. The model fit was significant (Likelihood Ratio χ² = 270.16, df = 6, p < 0.001), with a pseudo R² of 0.110, indicating that the included predictors account for a meaningful share of the variation in workplace transitions.

**Table 5:** Ordered Logit Model outputs.

Variable	Coefficient	Std. Error	z-value	p-value	Odds Ratio (Exp (Coef))	95% CI for OR
Intercept 1 (cut1)	−1.25	0.35	−3.57	<0.001	—	____
Intercept 2 (cut2)	0.85	0.36	2.36	0.018	—	____
Age (years)	−0.02	0.01	−2.00	0.045	0.98	0.96-1.0
Monthly Income (in 1000 SAR)	0.10	0.04	2.50	0.012	1.11	1.02–1.19
Marital Status (Married = 1)	−0.30	0.12	−2.50	0.013	0.74	0.59-0.94
Has Driver’s License (Yes = 1)	0.80	0.15	5.33	<0.001	2.23	1.66–2.99
Log-likelihood (null) −1220.45						
Log-likelihood (model) −1085.37						
Likelihood Ratio Chi-square 270.16						
Degrees of Freedom 6						
p-value (model fit) < 0.001						
Pseudo R-squared (McFadden) 0.110						
Akaike Information Criterion (AIC) 2184.74						
N = 890						

From Table 5, the OLM results highlight significant predictors across SEM levels. At the Individual Level, the coefficient for age was negative and significant (β = −0.02, p = 0.045), suggesting that younger women are more likely than older women to report workplace changes after gaining driving rights. This aligns with global evidence that younger cohorts adapt more quickly to new mobility opportunities. Monthly family income had a positive effect (β = 0.10, p = 0.012; OR = 1.11). Women from higher-income households were more likely to report workplace transitions, reflecting their ability to leverage mobility to access diverse job opportunities. It should be stated here that these findings should be interpreted as associations rather than evidence of causality (β = 0.80, p < 0.001; OR = 2.23). Licensed women were more than twice as likely to experience workplace transitions compared to non-licensed peers, highlighting the centrality of individual-level mobility capacity.

At the Interpersonal Level, being married was negatively associated with workplace change (β = −0.30, p = 0.013; OR = 0.74). Married women were significantly less likely to alter their employment, suggesting that family and childcare obligations, and spousal expectations constrain the extent to which driving rights translate into labour mobility. The final model primarily captures individual and interpersonal determinants of workplace transitions. Institutional/community and policy influences were explored descriptively and through the broader SEM interpretation rather than being directly estimated within the ordered logit specification.

From the table, it also appears that the confidence interval for age is very close to 1.00, which is consistent with its borderline statistical significance (p = 0.045). The confidence interval for driver's licence ownership indicates a robust and precisely estimated effect: women with a driver's licence had between 1.66 and 2.99 times higher odds of reporting workplace transitions than those without a licence. Because the confidence intervals for income, marital status, and licence ownership do not cross 1.00, these effects remain statistically significant at the 5% level.

In summary, the ordered logit results reinforce the multi-level dynamics of SEM in a number of ways. Individual capacity (younger age and higher income) promotes workplace mobility. Interpersonal constraints (marital status) moderate these opportunities. Community context (employment categories) influences how mobility benefits are distributed.

From the earlier descriptive analysis, it appears that policy reforms (lifting of the driving ban, Vision 2030 empowerment agenda) provide the enabling environment that makes these transitions possible. Overall, the findings demonstrate that while driving rights significantly increase women’s likelihood of workplace change, the degree of benefit varies depending on age, income, and household context, confirming the necessity of a multi-layered SEM perspective in understanding Saudi women’s workplace transitions.

## 6. Discussion of the results

The findings suggest that women’s mobility-related characteristics are associated with perceived workplace transitions through multiple influences captured by the Social Ecological Model (SEM). At the individual level, socio-demographic results (Table 1) show that the majority of respondents were in their prime working years (18–35), aligning with the ordered logit model results where age had a negative, significant effect (β = –0.02, p = 0.045; OR = 0.98). Younger women were more likely to change workplaces, reflecting greater willingness to leverage mobility reforms, while older women may be less inclined to report workplace transitions because they are more likely to have accumulated job-specific experience, established professional routines, and preferences for employment stability as they progress through their careers. In addition, the perceived risks associated with changing jobs may outweigh the potential benefits of mobility

Income was also significant (β = 0.10, p = 0.012; OR = 1.11), suggesting that higher-income women may be better able to convert mobility into workplace transitions because they possess complementary resources that facilitate labour market flexibility. These may include greater access to private vehicles and associated operating costs, the ability to secure childcare or domestic support, wider professional networks, and increased capacity to pursue employment opportunities located farther from home. Consequently, driving access may amplify existing socioeconomic advantages rather than benefiting all women equally.. Driver’s licence ownership emerged as the strongest predictor (β = 0.80, p < 0.001; OR = 2.23), suggesting that women with access to driving were more likely to report perceived workplace transitions and greater employment-related mobility. These findings are consistent with the notion that improved mobility may enhance women's perceived capacity to pursue workplace transitions; however, the present analysis cannot establish whether such perceptions necessarily translate into realised labour market outcomes. It is important to distinguish between mobility empowerment and realised labour market outcomes. While independent driving may increase women’s sense of autonomy, flexibility, and perceived access to opportunities, these forms of empowerment do not necessarily translate into actual workplace transitions, promotions, or long-term career progression. Such outcomes are influenced by a wider set of economic, institutional, and socio-cultural factors that were not directly measured in this study.

At the interpersonal level, marital status significantly constrained mobility outcomes (β = –0.30, p = 0.013; OR = 0.74). Married women were less likely to report workplace transitions, which may reflect the continuing influence of household responsibilities, childcare demands, and spousal expectations. These obligations can constrain the extent to which increased mobility is translated into employment-related mobility, even when independent driving is available.. This finding is consistent with prior research showing that gendered domestic obligations continue to moderate the effect of structural reforms on women’s employment in Saudi Arabia and comparable contexts [3,4,14]. These interpersonal dynamics illustrate that even when individual mobility is enhanced, household negotiations may limit the degree to which women act on new opportunities. It should be noted here that given the cross-sectional observational design, the ordered logit model estimates statistical associations between women's mobility-related characteristics and workplace transitions rather than causal effects.

More broadly, socio-cultural expectations surrounding women's roles within the family may shape perceptions of acceptable employment choices, commuting distances, and the feasibility of workplace transitions. Although the present study did not directly measure these cultural norms, the findings suggest that increased mobility alone may not automatically translate into equivalent employment-related mobility for all women. Rather, the benefits of the reform appear to be filtered through existing family structures and socially embedded expectations regarding caregiving and domestic responsibilities.

It should also be noted here that while employment category was positively associated with workplace change, with mid- and high-skilled women more likely to transition between jobs compared to those unemployed or in low-skilled positions (56.1% of highly skilled women expressed likelihood of change work place, while only 38% of Low-skilled Jobs respondents reported that they were likely to change). This indicates that sectoral diversity, professional labour markets, and organisational flexibility amplify the benefits of mobility. At first glance, this finding may appear contrary to expectations, as lower-skilled workers are often assumed to experience greater employment turnover. However, in the Saudi context, higher-skilled women may possess greater access to information, professional networks, and alternative employment opportunities that allow them to use newly acquired mobility to pursue career advancement, improved working conditions, or better remuneration. In contrast, women in lower-skilled occupations may face more constrained labour market opportunities, fewer transferable skills, and greater economic insecurity, making workplace transitions less feasible despite increased mobility. This suggests that driving reforms may disproportionately enhance the ability of women with greater human and social capital to capitalise on emerging employment opportunities.

It is also important to recognise that transport-related improvements may not fully overcome other structural barriers affecting women's employment-related mobility. Workplace segregation practices, organisational cultures, limited childcare availability, and the extent of family support may continue to restrict women's ability to pursue workplace transitions and career advancement, even when mobility constraints have been reduced.

At the policy level, Vision 2030 reforms were widely perceived as empowering, with 89% of respondents agreeing that these reforms facilitated women’s economic and social inclusion (Table 2). Although the ordered logit model cannot establish causality, the positive association between driver's licence ownership and perceived workplace transitions suggests that mobility reforms may create enabling conditions that support employment-related mobility among Saudi women. Importantly, increased mobility empowerment should not be equated with realised labour market outcomes. Rather, the findings indicate that the extent to which women perceive mobility as facilitating workplace transitions varies according to age, income, marital status, and broader social and institutional contexts. Overall, the SEM framework highlights that workplace transitions following mobility reforms are shaped by the interaction of individual characteristics, interpersonal relationships, and structural conditions, emphasising the need for complementary policies that address both transport-related and non-transport barriers to women's career mobility.

## 7. Conclusions, limitations, and suggested future research

This study examined the association between women's driving access and self-reported workplace mobility among Saudi women within the framework of the Social Ecological Model (SEM), using descriptive statistics and an Ordered Logit Model (OLM). The integration of SEM with econometric modelling provides a useful framework for understanding how mobility-related characteristics and socio-demographic factors are associated with perceived workplace transitions across multiple levels of influence.

Research Question 1 investigated whether driving access was associated with workplace transitions. The findings suggest a positive association between driver's licence ownership and self-reported workplace mobility, with licensed women reporting a greater likelihood of workplace transitions than women without licences. However, given the cross-sectional nature of the study, these associations should not be interpreted as evidence of a causal relationship between driving access and employment-related mobility.

Research Question 2 explored the role of socioeconomic and interpersonal factors. The analysis indicated that higher income was positively associated with perceived workplace transitions, whereas marital status was negatively associated with such transitions. These findings suggest that the extent to which women perceive themselves able to utilise mobility opportunities may vary according to individual resources and family circumstances.

By situating workplace transitions within the SEM framework, this study contributes to the literature by shifting attention from labour force participation to employment-related mobility among women already engaged in, or considering movement within, the labour market. The findings indicate that perceived workplace mobility is associated with both individual and interpersonal factors and that mobility empowerment should be distinguished from realised labour market outcomes.

Several limitations should be acknowledged. The study relied on self-reported survey responses and a cross-sectional design, which limits the ability to establish temporal ordering or causal relationships. Although the sample was large, it may not fully capture women who are less digitally connected or those living in underserved areas. Furthermore, important factors such as organisational practices, childcare availability, family support structures, and broader socio-cultural norms were not directly measured.

Future research should employ longitudinal or pre/post-reform designs to provide stronger evidence regarding how mobility relates to workplace transitions and career development over time. Panel data could help disentangle the potentially reciprocal relationships between licence ownership, household resources, and workplace mobility. In addition, qualitative approaches could deepen understanding of how family expectations, workplace environments, and social norms shape women's ability to translate increased mobility into realised employment outcomes.
