# Supplementary material for: Women's workplace transitions in saudi arabia following the driving reform: An ordered logit analysis
Source: PLoS One. 2026 Aug 31;21(8):e0354251. doi: 10.1371/journal.pone.0354251 (PMC13529003; doi:10.1371/journal.pone.0354251)
Supplement: Supplementary file 1 [file pone.0354251.s002.docx]

The authors would like to thank both reviewers for their thorough and considerate reviews. Their detailed comments and constructive recommendations have been invaluable in enhancing the scientific quality, methodological transparency, and overall presentation of the manuscript. We greatly appreciate the time and effort invested in reviewing our work and have addressed each comment carefully in the revised version.

**Reviewers' comments:**

**Reviewer #1: Comments to authors:
Comment 1**. Overall, the study addresses a highly relevant and timely topic aligned with major national reforms in Saudi Arabia, particularly Vision 2030. The research is well-motivated, and the findings contribute meaningfully to understanding the relationship between women’s mobility and employment outcomes. The use of both descriptive and inferential analysis, combined with a large and diverse sample, strengthens the empirical value of the work. The manuscript is well-structured and demonstrates a clear link between theoretical background, research questions, and practical implications.

**Response:** We thank the reviewer for a positive comment.

**Comment 2**. The abstract states that the study examines women’s employment opportunities; however, the results focus mainly on changes in workplace mobility among women who are already employed. To improve clarity and alignment, I suggest rephrasing the abstract and the interpretation of findings to emphasise job mobility and workplace transitions, rather than overall employment participation.

**Response**: We thank the reviewer for the comment. We agree on the suggested modification and the abstract has been modified accordingly. We have modified the abstract and replaced “employment transitions” and “access to job opportunities”, which can imply labour force entry, with “workplace transitions,” “career mobility,” and “employment-related mobility.” This clarifies that the study concerns mobility within employment rather than overall employment participation. Keywords have been modified accordingly as well.

**Comment 3**. The literature review is well-informed and relevant; however, it would benefit from improved synthesis and critical analysis. More concise summaries of cited studies, inclusion of recent research, clearer identification of gaps, and a tighter focus on the methodological relevance would enhance clarity and strengthen its academic contribution.

**Response:** Thank you for this insightful and constructive comment. In response, we have substantially revised the literature review to improve synthesis, strengthen the critical analysis, and more clearly position the contribution of the study. First, we reduced descriptive summaries of individual studies and reorganised the review around key thematic areas, including gender, mobility and labour market participation; socio-cultural and institutional barriers to women's employment-related mobility; and methodological approaches used to examine these relationships. Second, we incorporated recent and internationally relevant literature to situate the Saudi experience within broader debates on women's mobility, commuting, job search, and labour market participation. In particular, we integrated studies from Saudi Arabia and comparable contexts, including recent evidence on the employment effects of the Saudi driving reform (Abou Daher et al., 2025), women's mobility and labour supply in Pakistan (Field & Vyborny, 2022), and mobility challenges in public transport systems in Pakistan (Shoaib et al., 2025). We also incorporated recent work on legal reforms and women's employment in Saudi Arabia (Alharbi, 2025) and labour market participation in the MENA region (Fakih & Ghazalian, 2015). Third, we strengthened the identification of research gaps by emphasising that existing studies have predominantly examined labour force participation, empowerment, or broad employment outcomes, whereas little attention has been paid to employment-related mobility in the form of workplace transitions following the lifting of the driving ban. Finally, we clarified the methodological contribution of the study by explicitly highlighting the novelty of combining the Social Ecological Model with ordered logit modelling to examine multi-level determinants of workplace transitions among Saudi women. We believe these revisions improve the coherence, international relevance, and academic contribution of the literature review.

**Comment 4**. The methodology is clearly explained and appropriately aligned with the study objectives, with the ordered logit model offering a suitable approach for analysing the ordinal outcome. To further strengthen this section, it would be beneficial to provide additional details on the sampling strategy and to acknowledge any potential bias arising from the online mode of data collection. A brief justification for the use of a 3-point Likert scale, which may limit the nuance in respondents’ perceptions, is also recommended.

**Response:** Thank you for this constructive comment. In response, we have expanded the Methodology section to provide greater clarity regarding the sampling strategy, the potential limitations of online data collection, and the rationale for the use of a 3-point Likert scale. Specifically, we clarify that participants were recruited using a non-probability sampling approach that combined purposive dissemination through digital platforms with convenience sampling to maximise participation from Saudi women across diverse regions, professions, and socioeconomic backgrounds. We also acknowledge that the online mode of administration may have introduced selection bias by potentially underrepresenting women with limited internet access, lower digital literacy, older age groups, or those residing in remote areas. Finally, we provide a justification for the use of the 3-point Likert scale, noting that it was intentionally selected to simplify response choices, minimise respondent burden, and improve completion rates across a heterogeneous sample. While this approach may reduce the nuance captured in respondents' perceptions compared with scales containing more response categories, it was considered appropriate given the exploratory nature of the study and the broad demographic composition of the target population. These additions strengthen the transparency of the methodological approach and acknowledge its inherent limitations. These modifications have been added to the methodology section.

**Comment 5**: The research questions are clearly articulated and demonstrate strong alignment with the overall aim of examining the impact of driving on women’s employment mobility. RQ1 and RQ2 are well addressed through the findings of the ordered logit model, which provides appropriate empirical evidence in support of these dimensions. However, RQ3, concerning regional variation in mobility outcomes, is not substantiated by the results presented, as no geographic or rural–urban comparisons are included in the analysis. The author is advised to either revise or remove RQ3 to align with the available data, or alternatively incorporate regional-level analyses to support this research question.

**Response:** Thank you for this helpful observation; we agree with the reviewer. To ensure consistency between the research questions and the empirical evidence presented, we have removed RQ3 from the revised manuscript. The remaining research questions are directly aligned with the study objectives and are adequately addressed by the results. We appreciate the reviewer's suggestion, which has helped improve the clarity and coherence of the paper.

**Comment 6**. The inclusion of key demographic and socioeconomic variables is appreciated and contributes meaningfully to the analysis. As socio-cultural norms also play an important role in shaping women’s employment choices in the Saudi context, it may be beneficial to include a brief discussion of how cultural expectations and family responsibilities could influence the extent to which women are able to take advantage of increased mobility. This would help provide a more rounded interpretation of the findings.

**Response:** Thank you for this insightful comment. We agree that socio-cultural norms and family expectations are important factors influencing the extent to which women are able to benefit from increased mobility in the Saudi context. In response, we have strengthened the Discussion section to provide a more nuanced interpretation of the findings. Specifically, we elaborate on how household responsibilities, childcare obligations, spousal expectations, and prevailing gender norms may moderate the relationship between enhanced mobility and workplace transitions. While the driving reform has expanded women's mobility and increased opportunities for employment-related mobility, the extent to which these opportunities are realised continues to be shaped by family negotiations and cultural expectations surrounding women's domestic roles. We also emphasise this point in the conclusions and future research sections by noting that interpersonal and socio-cultural factors were not directly measured in the present study and recommending that future research employ qualitative approaches to explore these deeper family and cultural dynamics. We believe these revisions provide a more rounded and contextually grounded interpretation of the findings.

**Some minor suggestions:-
Comment 7**: It may be helpful to clarify the terminology used throughout, ensuring consistent use of “job mobility” or “workplace transition” rather than broader terms such as “employment participation,” so the interpretation remains aligned with the analysed dependent variable.

**Response:** Thank you for this helpful observation. We agree that consistent terminology is important to ensure that the interpretation of the findings accurately reflects the outcome variable analysed in this study. In response, we have carefully reviewed the manuscript and revised the terminology throughout to align with the dependent variable used in the ordered logit model. Specifically, broad terms such as "employment participation," "employment opportunities," and "labour market participation" have been replaced, where appropriate, with more precise terms including "job mobility," "employment-related mobility," "workplace transitions," and "workplace change." These revisions have been made in the title, abstract, introduction, research questions, results, discussion, conclusions, and keywords to improve conceptual clarity and avoid overstating the implications of the findings. We believe these changes strengthen the coherence of the manuscript and ensure that the interpretation remains consistent with the empirical analysis undertaken.

**Comment 8:** The narrative could benefit from briefly explaining why some results may differ from expected patterns (e.g., higher-skilled workers being more likely to change workplaces).
**Response:** Thank you for this helpful suggestion. We agree that providing additional interpretation for findings that may appear counterintuitive strengthens the discussion. In response, we have expanded the Discussion section to explain why higher-skilled women were found to be more likely to change workplaces following the driving reform. We note that, although lower-skilled workers are often assumed to exhibit higher job turnover, higher-skilled women in the Saudi context may have greater access to professional networks, labour market information, and alternative employment opportunities, enabling them to leverage increased mobility for career progression and improved employment conditions. Conversely, lower-skilled women may face more limited job options and greater economic constraints, reducing their ability to undertake workplace transitions. We believe this additional explanation provides a more nuanced interpretation of the findings and situates them within the broader Saudi labour market context.

**Comment 9**. Including confidence intervals alongside the odds ratios in the regression results would provide greater transparency regarding the precision of the estimates.
**Response:** Thank you for this helpful suggestion. We agree that reporting confidence intervals alongside odds ratios enhances transparency by providing information on the precision of the estimated effects. In response, we have revised the ordered logit regression table to include the 95% confidence intervals for all reported odds ratios. The confidence intervals confirm the robustness of the key findings, particularly the strong positive association between driver's licence ownership and workplace transitions. We believe that this addition improves the interpretability and statistical reporting of the results. We have also added a commentary on the results of the reported confidence intervals.

**Comment 10**. The study could be strengthened by explicitly acknowledging non-transport barriers (e.g., workplace segregation practices, limited childcare availability) that may continue to restrict job mobility despite improved driving access.

**Response:** Thank you for this valuable observation. We agree that improved driving access alone may not be sufficient to eliminate all barriers affecting women's employment-related mobility. In response, we have revised the Discussion and Conclusions sections to explicitly acknowledge that non-transport barriers, including workplace practices, organisational cultures, childcare responsibilities, and the availability of family support systems, may continue to constrain women's ability to translate enhanced mobility into workplace transitions and career advancement. While these factors were not directly measured in the present study, they are consistent with the Social Ecological Model, which recognises that individual behaviour and outcomes are shaped by broader interpersonal, institutional, and community contexts. We therefore emphasise that mobility reforms should be complemented by supportive workplace policies and family-oriented interventions to maximise their benefits. We have also highlighted these factors as important avenues for future research to provide a more comprehensive understanding of the determinants of women's employment-related mobility in Saudi Arabia.

**Reviewer #2: “Women’s Mobility and Employment Transitions in Saudi Arabia:** Assessing Labour Market Impacts Post-Driving Reform”

**General Comments**
The aim of the paper is to investigate the relationship between the 2018 lifting of the women’s driving ban in Saudi Arabia and women’s labor market mobility. This paper has important policy contribution related to gender economics, labor economics, transport policy, and Saudi Vision 2030. The study utilizes data from 890 Saudi women and applies descriptive statistics and an ordered logit model to examine the likelihood of workplace change after increased mobility. The paper has a solid base but by taking the below comments into consideration, I believe it can be made even stronger.

**Response:** We thank the reviewer for a positive comment.

**Comments on Abstract:**
1. Avoid using causal language such as “impact” with more cautious language with words such as “association,” “relationship,” or “perceived effect.”

**Response:** Thank you for your comment. We have replaced "impact," "influenced," "catalyst" with "association," "relationship," "suggest," and "perceived".

2. Clearly highlight whether the study is genuinely mixed-methods. If you intend on not using qualitative data or analyzing, it describes it as a quantitative survey study.

**Response**: Thank you for pointing this comment out. We clarified that the study is a quantitative survey study, rather than implying a mixed-methods design, as no qualitative data were collected or analysed
3. Report the ordered logit coefficients or main effect sizes

**Response:** Thank you for your valuable comment. We strengthened the reporting of findings by including the main effect size from the ordered logit model, reporting both the regression coefficient and odds ratio for the key predictor (driver's licence ownership: β = 0.80, OR = 2.23, *p* < 0.001).

4. Explain the role of secondary data in the econometric model

**Response:** Thank you for your valuable suggestion. We clarified the role of secondary data by specifying that official national statistics and policy reports were used to contextualise and interpret the findings at the institutional and policy levels and were not directly incorporated into the econometric estimation.

**Comments on Introduction and background:**

1. Separate the effect of driving from these broader reforms that came under the Vision of 2030 including labor market reforms, Saudization policies, changes in guardianship rules, expansion of female employment sectors, education gains, and social changes
2. Construct a clearer economic mechanism
3. Explain clearly how driving affects labor outcomes through lower commuting costs, lower dependence on male relatives or private drivers, greater schedule flexibility and access to jobs in areas poorly served by public transport
4. Make sure to also distinguish between employment participation, job change, job quality, occupational mobility and empowerment perceptions. Although these are very related yet they do not necessarily have the same outcome

**Response:** Thank you for these thoughtful comments. In response, we have substantially revised the Introduction to improve conceptual clarity and more carefully position the contribution of the study. First, we now explicitly distinguish the lifting of the driving ban from other concurrent Vision 2030 reforms, including labour market reforms, Saudization policies, changes in guardianship regulations, expansion of employment sectors available to women, improvements in educational attainment, and broader social transformations. We acknowledge that these reforms collectively shaped women's labour market experiences and that the present study does not attribute all observed changes solely to driving access.

Second, we have strengthened the theoretical and economic rationale underpinning the study by explicitly describing the mechanisms through which driving may relate to employment-related mobility. Specifically, we explain how independent driving can reduce commuting costs and travel time, decrease reliance on male relatives and private drivers, increase schedule flexibility, and expand access to workplaces located in areas with limited public transport provision. These mechanisms may facilitate workplace transitions and career mobility.

Third, we have revised the terminology throughout the Introduction to distinguish between different labour market outcomes, including labour force participation, workplace transitions, job quality, occupational mobility, and perceptions of empowerment. We clarify that the outcome examined in this study is employment-related mobility in the form of workplace transitions, rather than overall labour market participation or employment entry. We believe these revisions strengthen the conceptual framework, improve the interpretation of the findings, and more accurately position the study within the broader literature on women's mobility and labour market outcomes.

**Comments on Literature review:**
1. Avoid being too repetitive. Keep your ideas clear and straightforward
2. I would suggest introducing references that are more international related to gender, commuting, mobility, job search, and labor market participation
3. Clearly state the contribution of this study relative to the literature
4. Revise your references make sure they are complete and in correct format
5. Make sure all the references in this section are found in your reference list

**Response:** Thank you for these valuable comments. In response, we have substantially revised the literature review to improve its clarity, synthesis, and positioning within the broader scholarship. First, we reduced repetition and reorganised the review around key themes, including: (i) international evidence on gender, mobility, commuting, and labour market outcomes; (ii) the Saudi context and the implications of the driving reform; (iii) multi-level influences shaping employment-related mobility using the Social Ecological Model; and (iv) methodological approaches used to investigate these relationships. This restructuring allowed us to move beyond descriptive summaries of individual studies towards a more critical synthesis of the literature.

Second, we strengthened the international perspective by incorporating additional studies on women's mobility, job search, commuting constraints, and labour market participation from both regional and global contexts. These studies situate the Saudi experience within wider debates concerning transport disadvantage, gendered mobility, and employment opportunities.

Third, we clarified the specific contribution of this study relative to the existing literature. We emphasise that, unlike previous studies that have primarily examined labour force participation, empowerment, or broad employment outcomes, this study focuses specifically on employment-related mobility in the form of workplace transitions. We further highlight the novelty of combining the Social Ecological Model with ordered logit modelling to examine the multi-level determinants of workplace transitions following the lifting of the driving ban.

Finally, we carefully reviewed all citations and references to ensure consistency, completeness, and adherence to the journal's referencing style. We verified that all studies cited within the literature review are included in the reference list and that all reference details are complete and correctly formatted.

**Comments on Data collection and sample:**
1. Explain the sampling strategy clearly
2. Discuss recruitment methods including inclusion criteria, exclusion criteria, regional distribution, education distribution, employment status distribution and driving license and car ownership rates.
3. Discuss how missing variables were treated
4. Discuss online survey selection bias
5. I would suggest adding the questionnaire items in an appendix

**Response:** We thank the reviewer for this helpful suggestion. The methodology section has been expanded to provide greater detail regarding the non-probability purposive and convenience sampling strategy, recruitment procedures, inclusion and exclusion criteria, and the characteristics of the final sample. We have also clarified the handling of missing data and further acknowledged the potential for selection bias associated with online survey administration. Regarding questionnaire, it was developed and administered using the Google Forms platform and consists of a substantial number of items, including 27 attitudinal statements and several sections covering demographic, mobility, employment, and policy-related factors. Converting the full instrument into a format suitable for inclusion as an appendix would require extensive reformatting. To enhance transparency, we have expanded the Methods section to describe the questionnaire structure and content in greater details of its sections.

**Comments on Variables and measurements:**

1. Make sure that the model specification matches the reported table
2. Include all theoretically relevant variables and discuss why certain variables were excluded
3. Do not interpret variables that are not shown in the regression table

**Response:** We thank the reviewer for highlighting this issue. The methodology and results sections have been revised to ensure consistency between the ordered logit specification and the variables presented in Table 5. The revised manuscript now clearly states that the final model included age, monthly income, marital status, and driver's licence ownership. Variables considered within the broader SEM framework but not included in the final regression specification are now discussed descriptively rather than interpreted as regression findings. This revision improves transparency regarding variable selection and avoids attributing inferential results to variables not directly estimated in the model.

**Comments on Econometric method: ordered logit model:**
1. Include marginal effects or predicted probabilities.
2. Mention the number of observations in each model
3. Discuss potential endogeneity that can arise due to license ownership and car ownership
4. Clearly state that the model estimates association and not causal relationship

**Responses**:

We thank the reviewer for these constructive suggestions. The number of observations used in the ordered logit analysis (N = 890) has now been explicitly reported. We have also clarified throughout the manuscript that, given the cross-sectional observational design, the ordered logit model estimates associations rather than causal relationships. Furthermore, we acknowledge the potential for endogeneity, particularly with respect to driver's licence ownership and workplace transitions, as unobserved characteristics such as motivation, socioeconomic advantage, or career orientation may influence both. Finally, while marginal effects and predicted probabilities can enhance interpretation of ordered logit models, these were beyond the scope of the current revision and are acknowledged as a useful avenue for future research.

**Comments on Results:**
1. Make sure that the text and tables are consistent
2. Either present all 27 attitudinal items or discuss why certain selected items are shown
3. Consider adding a descriptive table for driving license ownership, car ownership, region, education and employment status
4. Add a full regression table with all variables listed in the methodology
5. Report predicted probabilities for the main driver’s license effect
6. Consider adding a robustness check with an alternative model to verify the reliability of your results
7. Revisit the interpretation of table 3 specifically

**Response:** We thank the reviewer for these constructive suggestions. We have revised the Results section to improve consistency between the text and tables and standardised the terminology used throughout. The attitudinal statements presented were selected because they most directly reflect the study objectives and the dimensions of the Social Ecological Model; this rationale has now been clarified. We have also expanded the descriptive presentation of the sample characteristics where possible to better characterise respondents' driving and employment profiles.

Furthermore, we have revised the interpretation of Table 3 to emphasise that these findings are descriptive and should not be interpreted causally. We acknowledge that predicted probabilities and robustness checks using alternative model specifications would provide additional insight; however, these analyses could not be undertaken during the current revision because the archived outputs of the final model, rather than the original dataset, were available. These points have now been acknowledged as limitations and recommendations for future research. Finally, we clarified that the ordered logit results reported correspond to the final calibrated model specification retained for interpretation.

**Comments on Discussions:**
1. Make sure that the discussion is tackled through associations and perceived employment mobility
2. Any claim that is not supported by the evidence provided it. Make sure it is removed or very briefly discussed
3. Clearly distinguish between “mobility empowerment” and actual labor market outcomes
4. Elaborate on why higher-income women may benefit more from driving access
5. Discuss why older and married women may be less likely to report workplace-change intentions

**Response:**

We thank the reviewer for these constructive comments. The Discussion section has been revised to consistently interpret the findings as statistical associations relating to perceived workplace transitions rather than causal labour market effects. Statements not directly supported by the empirical evidence were removed or reframed as possible explanations. We have also clarified the distinction between mobility empowerment and realised labour market outcomes, recognising that increased autonomy and perceived access to opportunities do not necessarily translate into actual career advancement. Additional discussion has been incorporated to explain why higher-income women may derive greater benefit from driving access and why older and married women may be less likely to report workplace-change intentions. These revisions strengthen the interpretation of the findings and ensure closer alignment between the evidence presented and the study objectives.

**Comments on Conclusion, limitations, and future research:**
1. In this section you discuss how urban women benefit more however your regression tables do not show regional effects
2. Make sure to discuss what is empirically shown rather than what is theoretically plausible
3. Mention that the findings suggest a positive association between driving access and self-reported workplace mobility
4. Avoid any causal claims
5. Mention that to ensure stronger evidence, papers should consider using pre/post data or longitudinal tracking

**Response:**

We thank the reviewer for these constructive comments. The Conclusion section has been revised to align more closely with the empirical evidence presented in the study. References to regional differences not estimated within the regression model have been removed, and the findings are consistently framed as associations rather than causal effects. The revised conclusion emphasises the positive association observed between driver's licence ownership and self-reported workplace mobility while distinguishing mobility empowerment from realised labour market outcomes. Finally, the future research section has been strengthened to recommend longitudinal and pre/post-reform approaches to provide stronger evidence regarding the relationship between mobility and workplace transitions over time.

**Comments on References:**
34. Abou Daher, C., Field, E., Swanson, K., & Vyborny, K. (2025). Drivers of change: Employment responses to the lifting of the Saudi female driving ban. American Economic Review, 115(9), 3248–3271.
35. Alharbi, H. A. (2025). Legal reforms of Saudi women’s right to work in the light of Vision 2030. Journal of Human Security, 21(1), 127–139.
36. Brant, R. (1990). Assessing proportionality in the proportional odds model for ordinal logistic regression. Biometrics, 46(4), 1171–1178.
37. Fakih, A., & Ghazalian, P. L. (2015). Female employment in MENA’s manufacturing sector: The implications of firm-related and national factors. Economic Change and Restructuring, 48(1), 37–69.
38. Fakih, A., Haimoun, N., & Sleiman, A. (2022). What drives demand for private tutoring in the Middle East and North Africa region? Evidence from a youth survey. African Development Review, 34(2), 268–279.
39. Field, E., & Vyborny, K. (2022). Women’s mobility and labor supply: Experimental evidence from Pakistan. ADB Economics Working Paper Series No. 655. Asian Development Bank.
40. Shoaib, A., Rana, I. A., & Adeel, M. (2025). Addressing women’s mobility challenges in the public transportation system: Evidence from Lahore, Pakistan. Transportation Research Part A: Policy and Practice, 195, Article 104419.
41. Shoaib, A., Rana, I. A., & Adeel, M. (2025). Addressing women’s mobility challenges in the public transportation system: Evidence from Lahore, Pakistan. Transportation Research Part A: Policy and Practice, 195, Article 104419

**Response:** We thank the reviewer for these valuable suggestions regarding the inclusion of additional references. The literature review has been substantially revised to incorporate the recommended international and regional studies related to gender, commuting, mobility, job search, and labour market participation. In particular, recent evidence examining the labour market implications of the Saudi driving reform (e.g., Abou Daher et al., 2025), women's mobility and labour supply in comparable contexts (e.g., Field & Vyborny, 2022), and transport-related barriers affecting women's employment opportunities in developing countries (e.g., Shoaib et al., 2025) has been integrated into the discussion. These additions strengthen the international positioning of the study, provide broader comparative perspectives, and help situate the Saudi experience within the wider literature on gender and mobility.

Furthermore, the literature review has been revised to improve synthesis and critical analysis, reduce repetition, clarify the methodological relevance of prior studies, and explicitly state the contribution of the present study. The reference list has also been carefully checked to ensure completeness, consistency of formatting, and correspondence between all in-text citations and the final reference list.
