# Supplementary material for: Women's workplace transitions in saudi arabia following the driving reform: An ordered logit analysis
Source: PLoS One. 2026 Aug 31;21(8):e0354251. doi: 10.1371/journal.pone.0354251 (PMC13529003; doi:10.1371/journal.pone.0354251)
Supplement: Peer review history

# Peer review: Decision Letter 0

**DOI:** 10.1371/journal.pone.0354251.r001

**Authors:** Nadine Campbell

5 May 2026

Dear Dr. Saleh,

Thank you for submitting your manuscript to PLOS ONE. After careful consideration, we feel that it has merit but does not fully meet PLOS ONE’s publication criteria as it currently stands. Therefore, we invite you to submit a revised version of the manuscript that addresses the points raised during the review process.

Please submit your revised manuscript by Jun 19 2026 11:59PM. If you will need more time than this to complete your revisions, please reply to this message or contact the journal office at plosone@plos.org. When you're ready to submit your revision, log on to https://www.editorialmanager.com/pone/ and select the 'Submissions Needing Revision' folder to locate your manuscript file.

- A letter that responds to each point raised by the academic editor and reviewer(s). You should upload this letter as a separate file labeled 'Response to Reviewers'.
- A marked-up copy of your manuscript that highlights changes made to the original version. You should upload this as a separate file labeled 'Revised Manuscript with Track Changes'.
- An unmarked version of your revised paper without tracked changes. You should upload this as a separate file labeled 'Manuscript'.

If you would like to make changes to your financial disclosure, please include your updated statement in your cover letter. Guidelines for resubmitting your figure files are available below the reviewer comments at the end of this letter.

If applicable, we recommend that you deposit your laboratory protocols in protocols.io to enhance the reproducibility of your results. Protocols.io assigns your protocol its own identifier (DOI) so that it can be cited independently in the future. For instructions, see: https://journals.plos.org/plosone/s/submission-guidelines#loc-laboratory-protocols. Additionally, PLOS ONE offers an option for publishing peer-reviewed Lab Protocol articles, which describe protocols hosted on protocols.io. Read more information on sharing protocols at https://plos.org/protocols?utm_medium=editorial-email&utm_source=authorletters&utm_campaign=protocols.

As the corresponding author, your ORCID ID is verified in the submission system and will appear in the published article. PLOS supports the use of ORCID, and we encourage all coauthors to register for an ORCID iD and use it as well. Please encourage your coauthors to verify their ORCID iD within the submission system before final acceptance, as unverified ORCID iDs will not appear in the published article. *Only*  the individual author can complete the verification step; PLOS staff *cannot*  verify ORCID iDs on behalf of authors.

We look forward to receiving your revised manuscript.

Kind regards,

Nadine Campbell, Ph.D.

Academic Editor

PLOS One

Journal Requirements:

When submitting your revision, we need you to address these additional requirements.

1. Please ensure that your manuscript meets PLOS ONE's style requirements, including those for file naming. The PLOS ONE style templates can be found at

https://journals.plos.org/plosone/s/file?id=wjVg/PLOSOne_formatting_sample_main_body.pdf and

https://journals.plos.org/plosone/s/file?id=ba62/PLOSOne_formatting_sample_title_authors_affiliations.pdf

<h2>2. Please provide additional details regarding participant consent. In the ethics statement in the Methods and online submission information, please ensure that you have specified (1) whether consent was informed and (2) what type you obtained (for instance, written or verbal, and if verbal, how it was documented and witnessed). If your study included minors, state whether you obtained consent from parents or guardians. If the need for consent was waived by the ethics committee, please include this information.</h2><h2>3. PLOS requires an ORCID iD for the corresponding author in Editorial Manager on papers submitted after December 6th, 2016. Please ensure that you have an ORCID iD and that it is validated in Editorial Manager. To do this, go to ‘Update my Information’ (in the upper left-hand corner of the main menu), and click on the Fetch/Validate link next to the ORCID field. This will take you to the ORCID site and allow you to create a new iD or authenticate a pre-existing iD in Editorial Manager.</h2><h2>4. Thank you for stating the following financial disclosure:</h2><h2>"the Deputyship for Research & Innovation, Ministry of Education in Saudi Arabia for funding this research work through project number WE-44-0031"      </h2><h2>Please state what role the funders took in the study.  If the funders had no role, please state: "The funders had no role in study design, data collection and analysis, decision to publish, or preparation of the manuscript."</h2><h2>If this statement is not correct you must amend it as needed.</h2><h2>Please include this amended Role of Funder statement in your cover letter; we will change the online submission form on your behalf.</h2><h2>5. Your ethics statement should only appear in the Methods section of your manuscript. If your ethics statement is written in any section besides the Methods, please move it to the Methods section and delete it from any other section. Please ensure that your ethics statement is included in your manuscript, as the ethics statement entered into the online submission form will not be published alongside your manuscript.</h2><h2>6. When completing the data availability statement of the submission form, you indicated that you will make your data available on acceptance. We strongly recommend all authors decide on a data sharing plan before acceptance, as the process can be lengthy and hold up publication timelines. Please note that, though access restrictions are acceptable now, your entire data will need to be made freely accessible if your manuscript is accepted for publication. This policy applies to all data except where public deposition would breach compliance with the protocol approved by your research ethics board. If you are unable to adhere to our open data policy, please kindly revise your statement to explain your reasoning and we will seek the editor's input on an exemption. Please be assured that, once you have provided your new statement, the assessment of your exemption will not hold up the peer review process.</h2>

7. If the reviewer comments include a recommendation to cite specific previously published works, please review and evaluate these publications to determine whether they are relevant and should be cited. There is no requirement to cite these works unless the editor has indicated otherwise.

8. Please review your reference list to ensure that it is complete and correct. If you have cited papers that have been retracted, please include the rationale for doing so in the manuscript text, or remove these references and replace them with relevant current references. Any changes to the reference list should be mentioned in the rebuttal letter that accompanies your revised manuscript. If you need to cite a retracted article, indicate the article’s retracted status in the References list and also include a citation and full reference for the retraction notice.

[Note: HTML markup is below. Please do not edit.]

Reviewers' comments:

Reviewer's Responses to Questions

**Comments to the Author**

1. Is the manuscript technically sound, and do the data support the conclusions?

Reviewer #1: Yes

Reviewer #2: Yes

**********

2. Has the statistical analysis been performed appropriately and rigorously?

Reviewer #1: Yes

Reviewer #2: Yes

**********

3. Have the authors made all data underlying the findings in their manuscript fully available?

The PLOS Data policy

Reviewer #1: Yes

Reviewer #2: Yes

**********

4. Is the manuscript presented in an intelligible fashion and written in standard English?

Reviewer #1: Yes

Reviewer #2: Yes

**********

Reviewer #1: Comments to authors:

1. Overall, the study addresses a highly relevant and timely topic aligned with major national reforms in Saudi Arabia, particularly Vision 2030. The research is well-motivated, and the findings contribute meaningfully to understanding the relationship between women’s mobility and employment outcomes. The use of both descriptive and inferential analysis, combined with a large and diverse sample, strengthens the empirical value of the work. The manuscript is well-structured and demonstrates a clear link between theoretical background, research questions, and practical implications.

2. The abstract states that the study examines women’s employment opportunities; however, the results focus mainly on changes in workplace mobility among women who are already employed. To improve clarity and alignment, I suggest rephrasing the abstract and the interpretation of findings to emphasise job mobility and workplace transitions, rather than overall employment participation.

3. The literature review is well-informed and relevant; however, it would benefit from improved synthesis and critical analysis. More concise summaries of cited studies, inclusion of recent research, clearer identification of gaps, and a tighter focus on the methodological relevance would enhance clarity and strengthen its academic contribution.

4. The methodology is clearly explained and appropriately aligned with the study objectives, with the ordered logit model offering a suitable approach for analysing the ordinal outcome. To further strengthen this section, it would be beneficial to provide additional details on the sampling strategy and to acknowledge any potential bias arising from the online mode of data collection. A brief justification for the use of a 3-point Likert scale, which may limit the nuance in respondents’ perceptions, is also recommended.

5. The research questions are clearly articulated and demonstrate strong alignment with the overall aim of examining the impact of driving on women’s employment mobility. RQ1 and RQ2 are well addressed through the findings of the ordered logit model, which provides appropriate empirical evidence in support of these dimensions. However, RQ3, concerning regional variation in mobility outcomes, is not substantiated by the results presented, as no geographic or rural–urban comparisons are included in the analysis. The author is advised to either revise or remove RQ3 to align with the available data, or alternatively incorporate regional-level analyses to support this research question.

6. The inclusion of key demographic and socioeconomic variables is appreciated and contributes meaningfully to the analysis. As socio-cultural norms also play an important role in shaping women’s employment choices in the Saudi context, it may be beneficial to include a brief discussion of how cultural expectations and family responsibilities could influence the extent to which women are able to take advantage of increased mobility. This would help provide a more rounded interpretation of the findings.

Some minor suggestions:-

7. It may be helpful to clarify the terminology used throughout, ensuring consistent use of “job mobility” or “workplace transition” rather than broader terms such as “employment participation,” so the interpretation remains aligned with the analysed dependent variable.

8. The narrative could benefit from briefly explaining why some results may differ from expected patterns (e.g., higher-skilled workers being more likely to change workplaces).

9. Including confidence intervals alongside the odds ratios in the regression results would provide greater transparency regarding the precision of the estimates.

10. The study could be strengthened by explicitly acknowledging non-transport barriers (e.g., workplace segregation practices, limited childcare availability) that may continue to restrict job mobility despite improved driving access.

Reviewer #2: “Women’s Mobility and Employment Transitions in Saudi Arabia: Assessing Labour Market Impacts Post-Driving Reform”

General Comments

The aim of the paper is to investigate the relationship between the 2018 lifting of the women’s driving ban in Saudi Arabia and women’s labor market mobility. This paper has important policy contribution related to gender economics, labor economics, transport policy, and Saudi Vision 2030. The study utilizes data from 890 Saudi women and applies descriptive statistics and an ordered logit model to examine the likelihood of workplace change after increased mobility. The paper has a solid base but by taking the below comments into consideration, I believe it can be made even stronger.

Abstract

1. Avoid using causal language such as “impact” with more cautious language with words such as “association,” “relationship,” or “perceived effect.”

2. Clearly highlight whether the study is genuinely mixed-methods. If you intend on not using qualitative data or analyzing, it describes it as a quantitative survey study

3. Report the ordered logit coefficients or main effect sizes

4. Explain the role of secondary data in the econometric model

Introduction and background

1. Separate the effect of driving from these broader reforms that came under the Vision of 2030 including labor market reforms, Saudization policies, changes in guardianship rules, expansion of female employment sectors, education gains, and social changes

2. Construct a clearer economic mechanism

3. Explain clearly how driving affects labor outcomes through lower commuting costs, lower dependence on male relatives or private drivers, greater schedule flexibility and access to jobs in areas poorly served by public transport

4. Make sure to also distinguish between employment participation, job change, job quality, occupational mobility and empowerment perceptions. Although these are very related yet they do not necessarily have the same outcome

Literature review

1. Avoid being too repetitive. Keep your ideas clear and straightforward

2. I would suggest introducing references that are more international related to gender, commuting, mobility, job search, and labor market participation

3. Clearly state the contribution of this study relative to the literature

4. Revise your references make sure they are complete and in correct format

5. Make sure all the references in this section are found in your reference list

Data collection and sample

1. Explain the sampling strategy clearly

2. Discuss recruitment methods including inclusion criteria, exclusion criteria, regional distribution, education distribution, employment status distribution and driving license and car ownership rates.

3. Discuss how missing variables were treated

4. Discuss online survey selection bias

5. I would suggest adding the questionnaire items in an appendix

Variables and measurement

1. Make sure that the model specification matches the reported table

2. Include all theoretically relevant variables and discuss why certain variables were excluded

3. Do not interpret variables that are not shown in the regression table

Econometric method: ordered logit model

1. Include marginal effects or predicted probabilities.

2. Mention the number of observations in each model

3. Discuss potential endogeneity that can arise due to license ownership and car ownership

4. Clearly state that the model estimates association and not causal relationship

Results

1. Make sure that the text and tables are consistent

2. Either present all 27 attitudinal items or discuss why certain selected items are shown

3. Consider adding a descriptive table for driving license ownership, car ownership, region, education and employment status

4. Add a full regression table with all variables listed in the methodology

5. Report predicted probabilities for the main driver’s license effect

6. Consider adding a robustness check with an alternative model to verify the reliability of your results

7. Revisit the interpretation of table 3 specifically

Discussion

1. Make sure that the discussion is tackled through associations and perceived employment mobility

2. Any claim that is not supported by the evidence provided it. Make sure it is removed or very briefly discussed

3. Clearly distinguish between “mobility empowerment” and actual labor market outcomes

4. Elaborate on why higher-income women may benefit more from driving access

5. Discuss why older and married women may be less likely to report workplace-change intentions

Conclusion, limitations, and future research

1. In this section you discuss how urban women benefit more however your regression tables do not show regional effects

2. Make sure to discuss what is empirically shown rather than what is theoretically plausible

3. Mention that the findings suggest a positive association between driving access and self-reported workplace mobility

4. Avoid any causal claims

5. Mention that to ensure stronger evidence, papers should consider using pre/post data or longitudinal tracking

References

Abou Daher, C., Field, E., Swanson, K., & Vyborny, K. (2025). Drivers of change: Employment responses to the lifting of the Saudi female driving ban. American Economic Review, 115(9), 3248–3271.

Alharbi, H. A. (2025). Legal reforms of Saudi women’s right to work in the light of Vision 2030. Journal of Human Security, 21(1), 127–139.

Brant, R. (1990). Assessing proportionality in the proportional odds model for ordinal logistic regression. Biometrics, 46(4), 1171–1178.

Fakih, A., & Ghazalian, P. L. (2015). Female employment in MENA’s manufacturing sector: The implications of firm-related and national factors. Economic Change and Restructuring, 48(1), 37–69.

Fakih, A., Haimoun, N., & Sleiman, A. (2022). What drives demand for private tutoring in the Middle East and North Africa region? Evidence from a youth survey. African Development Review, 34(2), 268–279.

Field, E., & Vyborny, K. (2022). Women’s mobility and labor supply: Experimental evidence from Pakistan. ADB Economics Working Paper Series No. 655. Asian Development Bank.

Shoaib, A., Rana, I. A., & Adeel, M. (2025). Addressing women’s mobility challenges in the public transportation system: Evidence from Lahore, Pakistan. Transportation Research Part A: Policy and Practice, 195, Article 104419.

Shoaib, A., Rana, I. A., & Adeel, M. (2025). Addressing women’s mobility challenges in the public transportation system: Evidence from Lahore, Pakistan. Transportation Research Part A: Policy and Practice, 195, Article 104419

**********

what does this mean?). If published, this will include your full peer review and any attached files.

If you choose “no”, your identity will remain anonymous but your review may still be made public.

**Do you want your identity to be public for this peer review?**  For information about this choice, including consent withdrawal, please see our Privacy Policy

Reviewer #1: **Yes:**  Dr. Anamika Moktan

Reviewer #2: No

**********

[NOTE: If reviewer comments were submitted as an attachment file, they will be attached to this email and accessible via the submission site. Please log into your account, locate the manuscript record, and check for the action link "View Attachments". If this link does not appear, there are no attachment files.]

To ensure your figures meet our technical requirements, please review our figure guidelines: https://journals.plos.org/plosone/s/figures

You may also use PLOS’s free figure tool, NAAS, to help you prepare publication quality figures: https://journals.plos.org/plosone/s/figures#loc-tools-for-figure-preparation.

NAAS will assess whether your figures meet our technical requirements by comparing each figure against our figure specifications.

# Author comment: Author response to Decision Letter 1

**DOI:** 10.1371/journal.pone.0354251.r002

30 Jun 2026

The authors would like to thank both reviewers for their thorough and considerate reviews. Their detailed comments and constructive recommendations have been invaluable in enhancing the scientific quality, methodological transparency, and overall presentation of the manuscript. We greatly appreciate the time and effort invested in reviewing our work and have addressed each comment carefully in the revised version.

Reviewers' comments:

Reviewer #1: Comments to authors:

Comment 1. Overall, the study addresses a highly relevant and timely topic aligned with major national reforms in Saudi Arabia, particularly Vision 2030. The research is well-motivated, and the findings contribute meaningfully to understanding the relationship between women’s mobility and employment outcomes. The use of both descriptive and inferential analysis, combined with a large and diverse sample, strengthens the empirical value of the work. The manuscript is well-structured and demonstrates a clear link between theoretical background, research questions, and practical implications.

Response: We thank the reviewer for a positive comment.

Comment 2. The abstract states that the study examines women’s employment opportunities; however, the results focus mainly on changes in workplace mobility among women who are already employed. To improve clarity and alignment, I suggest rephrasing the abstract and the interpretation of findings to emphasise job mobility and workplace transitions, rather than overall employment participation.

Response: We thank the reviewer for the comment. We agree on the suggested modification and the abstract has been modified accordingly. We have modified the abstract and replaced “employment transitions” and “access to job opportunities”, which can imply labour force entry, with “workplace transitions,” “career mobility,” and “employment-related mobility.” This clarifies that the study concerns mobility within employment rather than overall employment participation. Keywords have been modified accordingly as well.

Comment 3. The literature review is well-informed and relevant; however, it would benefit from improved synthesis and critical analysis. More concise summaries of cited studies, inclusion of recent research, clearer identification of gaps, and a tighter focus on the methodological relevance would enhance clarity and strengthen its academic contribution.

Response: Thank you for this insightful and constructive comment. In response, we have substantially revised the literature review to improve synthesis, strengthen the critical analysis, and more clearly position the contribution of the study. First, we reduced descriptive summaries of individual studies and reorganised the review around key thematic areas, including gender, mobility and labour market participation; socio-cultural and institutional barriers to women's employment-related mobility; and methodological approaches used to examine these relationships. Second, we incorporated recent and internationally relevant literature to situate the Saudi experience within broader debates on women's mobility, commuting, job search, and labour market participation. In particular, we integrated studies from Saudi Arabia and comparable contexts, including recent evidence on the employment effects of the Saudi driving reform (Abou Daher et al., 2025), women's mobility and labour supply in Pakistan (Field & Vyborny, 2022), and mobility challenges in public transport systems in Pakistan (Shoaib et al., 2025). We also incorporated recent work on legal reforms and women's employment in Saudi Arabia (Alharbi, 2025) and labour market participation in the MENA region (Fakih & Ghazalian, 2015). Third, we strengthened the identification of research gaps by emphasising that existing studies have predominantly examined labour force participation, empowerment, or broad employment outcomes, whereas little attention has been paid to employment-related mobility in the form of workplace transitions following the lifting of the driving ban. Finally, we clarified the methodological contribution of the study by explicitly highlighting the novelty of combining the Social Ecological Model with ordered logit modelling to examine multi-level determinants of workplace transitions among Saudi women. We believe these revisions improve the coherence, international relevance, and academic contribution of the literature review.

Comment 4. The methodology is clearly explained and appropriately aligned with the study objectives, with the ordered logit model offering a suitable approach for analysing the ordinal outcome. To further strengthen this section, it would be beneficial to provide additional details on the sampling strategy and to acknowledge any potential bias arising from the online mode of data collection. A brief justification for the use of a 3-point Likert scale, which may limit the nuance in respondents’ perceptions, is also recommended.

Response: Thank you for this constructive comment. In response, we have expanded the Methodology section to provide greater clarity regarding the sampling strategy, the potential limitations of online data collection, and the rationale for the use of a 3-point Likert scale. Specifically, we clarify that participants were recruited using a non-probability sampling approach that combined purposive dissemination through digital platforms with convenience sampling to maximise participation from Saudi women across diverse regions, professions, and socioeconomic backgrounds. We also acknowledge that the online mode of administration may have introduced selection bias by potentially underrepresenting women with limited internet access, lower digital literacy, older age groups, or those residing in remote areas. Finally, we provide a justification for the use of the 3-point Likert scale, noting that it was intentionally selected to simplify response choices, minimise respondent burden, and improve completion rates across a heterogeneous sample. While this approach may reduce the nuance captured in respondents' perceptions compared with scales containing more response categories, it was considered appropriate given the exploratory nature of the study and the broad demographic composition of the target population. These additions strengthen the transparency of the methodological approach and acknowledge its inherent limitations. These modifications have been added to the methodology section.

Comment 5: The research questions are clearly articulated and demonstrate strong alignment with the overall aim of examining the impact of driving on women’s employment mobility. RQ1 and RQ2 are well addressed through the findings of the ordered logit model, which provides appropriate empirical evidence in support of these dimensions. However, RQ3, concerning regional variation in mobility outcomes, is not substantiated by the results presented, as no geographic or rural–urban comparisons are included in the analysis. The author is advised to either revise or remove RQ3 to align with the available data, or alternatively incorporate regional-level analyses to support this research question.

Response: Thank you for this helpful observation; we agree with the reviewer. To ensure consistency between the research questions and the empirical evidence presented, we have removed RQ3 from the revised manuscript. The remaining research questions are directly aligned with the study objectives and are adequately addressed by the results. We appreciate the reviewer's suggestion, which has helped improve the clarity and coherence of the paper.

Comment 6. The inclusion of key demographic and socioeconomic variables is appreciated and contributes meaningfully to the analysis. As socio-cultural norms also play an important role in shaping women’s employment choices in the Saudi context, it may be beneficial to include a brief discussion of how cultural expectations and family responsibilities could influence the extent to which women are able to take advantage of increased mobility. This would help provide a more rounded interpretation of the findings.

Response: Thank you for this insightful comment. We agree that socio-cultural norms and family expectations are important factors influencing the extent to which women are able to benefit from increased mobility in the Saudi context. In response, we have strengthened the Discussion section to provide a more nuanced interpretation of the findings. Specifically, we elaborate on how household responsibilities, childcare obligations, spousal expectations, and prevailing gender norms may moderate the relationship between enhanced mobility and workplace transitions. While the driving reform has expanded women's mobility and increased opportunities for employment-related mobility, the extent to which these opportunities are realised continues to be shaped by family negotiations and cultural expectations surrounding women's domestic roles. We also emphasise this point in the conclusions and future research sections by noting that interpersonal and socio-cultural factors were not directly measured in the present study and recommending that future research employ qualitative approaches to explore these deeper family and cultural dynamics. We believe these revisions provide a more rounded and contextually grounded interpretation of the findings.

Some minor suggestions:-

Comment 7: It may be helpful to clarify the terminology used throughout, ensuring consistent use of “job mobility” or “workplace transition” rather than broader terms such as “employment participation,” so the interpretation remains aligned with the analysed dependent variable.

Response: Thank you for this helpful observation. We agree that consistent terminology is important to ensure that the interpretation of the findings accurately reflects the outcome variable analysed in this study. In response, we have carefully reviewed the manuscript and revised the terminology throughout to align with the dependent variable used in the ordered logit model. Specifically, broad terms such as "employment participation," "employment opportunities," and "labour market participation" have been replaced, where appropriate, with more precise terms including "job mobility," "employment-related mobility," "workplace transitions," and "workplace change." These revisions have been made in the title, abstract, introduction, research questions, results, discussion, conclusions, and keywords to improve conceptual clarity and avoid overstating the implications of the findings. We believe these changes strengthen the coherence of the manuscript and ensure that the interpretation remains consistent with the empirical analysis undertaken.

Comment 8: The narrative could benefit from briefly explaining why some results may differ from expected patterns (e.g., higher-skilled workers being more likely to change workplaces).

Response: Thank you for this helpful suggestion. We agree that providing additional interpretation for findings that may appear counterintuitive strengthens the discussion. In response, we have expanded the Discussion section to explain why higher-skilled women were found to be more likely to change workplaces following the driving reform. We note that, although lower-skilled workers are often assumed to exhibit higher job turnover, higher-skilled women in the Saudi context may have greater access to professional networks, labour market information, and alternative employment opportunities, enabling them to leverage increased mobility for career progression and improved employment conditions. Conversely, lower-skilled women may face more limited job options and greater economic constraints, reducing their ability to undertake workplace transitions. We believe this additional explanation provides a more nuanced interpretation of the findings and situates them within the broader Saudi labour market context.

Comment 9. Including confidence intervals alongside the odds ratios in the regression results would provide greater transparency regarding the precision of the estimates.

Response: Thank you for this helpful suggestion. We agree that reporting confidence intervals alongside odds ratios enhances transparency by providing information on the precision of the estimated effects. In response, we have revised the ordered logit regression table to include the 95% confidence intervals for all reported odds ratios. The confidence intervals confirm the robustness of the key findings, particularly the strong positive association between driver's licence ownership and workplace transitions. We believe that this addition improves the interpretability and statistical reporting of the results. We have also added a commentary on the results of the reported confidence intervals.

Comment 10. The study could be strengthened by explicitly acknowledging non-transport barriers (e.g., workplace segregation practices, limited childcare availability) that may continue to restrict job mobility despite improved driving access.

Response: Thank you for this valuable observation. We agree that improved driving access alone may not be sufficient to eliminate all barriers affecting women's employment-related mobility. In response, we have revised the Discussion and Conclusions sections to explicitly acknowledge that non-transport barriers, including workplace practices, organisational cultures, childcare responsibilities, and the availability of family support systems, may continue to constrain women's ability to translate enhanced mobility into workplace transitions and career advancement. While these factors were not directly measured in the present study, they are consistent with the Social Ecological Model, which recognises that individual behaviour and outcomes are shaped by broader interpersonal, institutional, and community contexts. We therefore emphasise that mobility reforms should be complemented by supportive workplace policies and family-oriented interventions to maximise their benefits. We have also highlighted these factors as important avenues for future research to provide a more comprehensive understanding of the determinants of women's employment-related mobility in Saudi Arabia.

Reviewer #2: “Women’s Mobility and Employment Transitions in Saudi Arabia: Assessing Labour Market Impacts Post-Driving Reform”

General Comments

The aim of the paper is to investigate the relationship between the 2018 lifting of the women’s driving ban in Saudi Arabia and women’s labor market mobility. This paper has important policy contribution related to gender economics, labor economics, transport policy, and Saudi Vision 2030. The study utilizes data from 890 Saudi women and applies descriptive statistics and an ordered logit model to examine the likelihood of workplace change after increased mobility. The paper has a solid base but by taking the below comments into consideration, I believe it can be made even stronger.

Response: We thank the reviewer for a positive comment.

Comments on Abstract:

1. Avoid using causal language such as “impact” with more cautious language with words such as “association,” “relationship,” or “perceived effect.”

Response: Thank you for your comment. We have replaced "impact," "influenced," "catalyst" with "association," "relationship," "suggest," and "perceived".

2. Clearly highlight whether the study is genuinely mixed-methods. If you intend on not using qualitative data or analyzing, it describes it as a quantitative survey study.

Response: Thank you for pointing this comment out. We clarified that the study is a quantitative survey study, rather than implying a mixed-methods design, as no qualitative data were collected or analysed

3. Report the ordered logit coefficients or main effect sizes

Response: Thank you for your valuable comment. We strengthened the reporting of findings by including the main effect size from the ordered logit model, reporting both the regression coefficient and odds ratio for the key predictor (driver's licence ownership: β = 0.80, OR = 2.23, p < 0.001).

4. Explain the role of secondary data in the econometric model

Response: Thank you for your valuable suggestion. We clarified the role of secondary data by specifying that official national statistics and policy r

Attachment Submitted filename: Responses to reviewers comments-Saleh.docx

# Editor report: Decision Letter 1

**DOI:** 10.1371/journal.pone.0354251.r003

**Authors:** Nadine Campbell

7 Jul 2026

<p>Women's Workplace Transitions in Saudi Arabia Following the Driving Reform: An Ordered Logit Analysis

PONE-D-25-50594R1

Dear Dr. Saleh,

We’re pleased to inform you that your manuscript has been judged scientifically suitable for publication and will be formally accepted for publication once it meets all outstanding technical requirements.

Within one week, you’ll receive an e-mail detailing the required amendments. When these have been addressed, you’ll receive a formal acceptance letter and your manuscript will be scheduled for publication.

An invoice will be generated when your article is formally accepted. Please note, if your institution has a publishing partnership with PLOS and your article meets the relevant criteria, all or part of your publication costs will be covered. Please make sure your user information is up-to-date by logging into Editorial Manager at Editorial Manager® and clicking the ‘Update My Information' link at the top of the page. For questions related to billing, please contact billing support.

If your institution or institutions have a press office, please notify them about your upcoming paper to help maximize its impact. If they’ll be preparing press materials, please inform our press team as soon as possible -- no later than 48 hours after receiving the formal acceptance. Your manuscript will remain under strict press embargo until 2 pm Eastern Time on the date of publication. For more information, please contact onepress@plos.org.

Kind regards,

Nadine Campbell, Ph.D.

Academic Editor

PLOS One

Additional Editor Comments (optional):

Reviewers' comments:
